# The added value of the artificial intelligence patient-reported experience measure (AI-PREM tool) in clinical practise: Deployment in a vestibular schwannoma care pathway

**DOI:** 10.1016/j.pecinn.2023.100204

**Published:** 2023-08-30

**Authors:** O.M. Neve, M.M. van Buchem, M. Kunneman, P.P.G. van Benthem, H. Boosman, E.F. Hensen

**Affiliations:** a Department of Otorhinolaryngology and Head and Neck Surgery, Leiden University Medical Centre, the Netherlands; b Information Technology & Digital Innovation Department, Leiden University Medical Centre, the Netherlands; c Knowledge and Evaluation Research Unit, Mayo Clinic, Rochester, MN, United States of America; d Department of Biomedical Data Sciences, Leiden University Medical Centre, Leiden, the Netherlands; e Morgens consultancy, Leiden, the Netherlands

**Keywords:** Patient experience, Quality of care, Patient centredness, Artificial intelligence, Vestibular schwannoma

## Abstract

**Objectives:**

Patient-reported experience measures (PREMs) can be used for the improvement of quality of care. In this study, the outcome of an open-ended question PREM combined with computer-assisted analysis is compared to the outcome of a closed-ended PREM questionnaire.

**Methods:**

This survey study assessed the outcome of the open-ended questionnaire PREM and a close-ended question PREM of patients with unilateral vestibular schwannoma in a tertiary vestibular schwannoma expert centre.

**Results:**

The open-ended questions PREM, consisting of five questions, was completed by 507 participants and resulted in 1508 positive and 171 negative comments, categorised into 27 clusters. The close-ended questions PREM results were mainly positive (overall experience graded as 8/10), but did not identify specific action points. Patients who gave high overall scores (>8) on the close-ended question provided points for improvement in the open-ended question PREM, which would have been missed using the close-ended questions only.

**Conclusions:**

Compared to the close-ended question PREM, the open-ended question PREM provides more detailed and specific information about the patient experience in the vestibular schwannoma care pathway.

**Innovation:**

Automated analysis of feedback with the open-ended question PREM revealed relevant insights and identified topics for targeted quality improvement, whereas the close-ended PREM did not.

## 1 Introduction

Patient experiences are important indicators of the quality of care. According to the national health service (NHS) policies, patient experiences reflect the compassion, dignity and respect for patients during health care delivery [1,2]. Moreover, these experiences may hold important insights for quality improvement [3]. Adequate tools to survey and analyse patient experiences are therefore essential. Patient experiences can be measured using patient-reported experience measures (PREMs), usually in the form of questionnaires [4].

PREMs may be considered subjective, but a positive association between PREM results and other quality domains has been reported [5]. PREM scores are positively but weakly associated with patient safety and clinical effectiveness, which suggests that improving patient experiences may enhance the overall quality of care [6,7]. Today, there are many different PREMs in use; most of them are disease or treatment specific and consist predominantly of closed-ended questions [[8], [9], [10], [11], [12]]. Some generic PREMs have been developed and are used to benchmark hospitals at a regional, national or international level [[13], [14], [15], [16], [17]].

The increased use of PREMs is incentivised by regulatory bodies in the United Kingdom and United States of America. Frequently, PREMs are collected and analysed but translating the results into changes in clinical practice remains challenging due to organizational, professional and data-related barriers [[18], [19], [20]]. The lack of a quality improvement infrastructure is one of these barriers [20]. Furthermore, patient experiences are not always adopted by clinicians, because the PREM results do not provide insights relevant to their daily workflow, or because the feedback is not specific enough to allow translation into concrete action points [3,19]. When PREM results are not translated into clear and actionable points of improvement for care providers, PREMs risk to be viewed as measurement for the sake of measurement rather than as valuable instruments for improving the underlying care [21].

In contrast to closed-ended questions that steer a patient's feedback to a specific topic, open-ended questions enable patients to provide feedback on all aspects of care that matter to them [22]. This feature makes open-ended questions more patient-centred and yields more specific information, facilitating concrete quality improvement measures [23]. However, the analysis of free-text answers is time-consuming and too laborious to use in clinical practice [23,24].

Artificial intelligence (AI) techniques are able to automatically detect the topics and sentiment of patients' free text comments and help identify actionable insights out of PREMs [25,26].

Currently used PREMs are not ideally suited for the full exploitation of the potential of AI-techniques. First, current questionnaires often contain questions with a sentiment comprised in the question itself. (e,g: ‘what went remarkably well during your stay?’ or ‘what could we improve?’). In addition, questions such as these invite short, monosyllabic answers, which are difficult to categorize [25]. To tackle these problems several modifications to commonly used PREMs are needed. A new AI-PREM tool has been developed and validated by Van Buchem et al. [27]^,^ with open-ended generic questions (i.e., not targeted at a specific disease, care pathway, department or healthcare centre) and suited for computer analysis by removing the sentiment from the question. The questions were focused on the Picker dimensions of patient-centred care to reduce the number of topics in an answer (e.g., What did you think about the information provision?) [27].

The primary aim of this study was to determine the added value of the AI-PREM tool compared to a conventional PREM with respect to identification of actionable points for quality improvement. The secondary aim was to assess the influence of socio-demographic determinants on AI-PREM completion and results. To do so, we have deployed the AI-PREM in a vestibular schwannoma integrated practice unit (IPU) in a vestibular schwannoma expert centre in the Netherlands.

## 2 Methods

### 2.1 Context

Vestibular schwannomas are rare benign intracranial tumours, which typically cause hearing loss, tinnitus and balance disorders. A majority (52–78%) of the tumours is non-progressive. In these cases active surveillance with prolonged follow-up is usually the management strategy of choice [28]. In case of very large or progressive tumours, surgery or radiotherapy is indicated to prevent future complications such as brain stem compression. After active therapy, prolonged follow-up is warranted to detect residual or recurrent disease. Because of the long follow-up required (with or without active treatment) and near to normal life expectancy with adequate management of the tumour, patients with a vestibular schwannoma often accumulate extensive experience with healthcare professionals and centres.

### 2.2 Design

This descriptive case study evaluated the outcomes of an open-ended question PREM and a close-ended question PREM employed in a vestibular schwannoma IPU. A non-responder analysis was performed, the outcomes of both PREM were analysed, and the ceiling effect was evaluated in a direct comparison. In addition, the interpretation and the selection of actionable points of improvement by the IPU team based on these outcomes was observed. The process to come from PREM results to actionable points of improvement is reported.

The study was performed at the Leiden University Medical Centre, a tertiary university hospital, and expert centre for vestibular schwannomas in The Netherlands. At our centre, patient care is organized in an IPU, including otorhinolaryngologists, neurosurgeons, radiation oncologists and radiologists. The combination of chronic care and the multidisciplinary organization in an IPU are ideal to investigate the added value of AI-PREM for quality improvement.

### 2.3 Participants

This study was part of larger study on long term quality of life in vestibular schwannoma patients. For longitudinal follow-up patients who participated in 2014 in a cross-sectional survey on quality of life in vestibular schwannoma patients were re-invited for participation [29]. Using this patient group allowed the analysis of non-responders based on the data collected in 2014. In 2014, all consecutive patients who were diagnosed or treated for a unilateral vestibular schwannoma since 2003 at the IPU were eligible for inclusion. Patients under 18 years, patients with insufficient proficiency in the Dutch language to complete the questionnaires or patients with other skull base pathologies were excluded. Data collection took place between June and September 2020. The local medical research and ethics committee has waived the necessity for medical ethical approval under Dutch law and approved the study regarding data handling and privacy regulations (N19.112).

### 2.4 Data collection

After providing informed consent, patients were asked to complete two validated PREM questionnaires either electronically or on paper. First, participants completed the AI-PREM, consisting of five open-ended questions about their experiences with the care delivery [27]. The five questions (Box 1) addressed the following themes: information provision, personal approach, collaboration, organization and other experiences, and were based on the Picker dimensions of patient-centred care [13,30]. The free-text answers were analysed using natural language processing techniques, which divided the free-text answers into clusters of positive and negative comments. These techniques are described in more detail by Van Buchem et al. [27] The output of the AI-PREM are clusters of positive and negative comments for each of the five questions. The output was accessible in a easily intelligible dashboard. This dashboard was able to show the thematically clustered patient feedback, differentiate negative from positive clusters, and quantify the number of comments per thematic cluster. In addition, the IPU team could access the full individual patient comments the clusters were based on (as raw text). .

Box 1 **Questions AI-PREM [30]**

- Q1: How was the provided information?
- Q2: How was the personal approach?
- Q3: How was the collaboration between healthcare professionals?
- Q4: How was the organization of care?
- Q5: What else would you like to share about your experience?

Alt-text: Box 1

Second, participants completed the Patient Experience Monitor (PEM) consisting of fifteen closed-ended questions about the patient's experience [14]; The PEM outcomes are proportions of patients which answered with a certain multiple choice option. For example, the proportion of the total number of respondents that trusted their physician fully.

Third, patients were asked to complete a disease-specific quality of life questionnaire of 26 items, the Penn Acoustic Neuroma Quality Of Life (PANQOL) [31,32]. Furthermore, demographic information (sex, age and education level) was acquired. Statistics Netherlands' (CBS) definition for low, middle and high education level was used, which follows the international standard classification of education [33].

Treatment modality, tumour size at baseline, and time since diagnosis were acquired from the electronic patient records. Treatment was coded as either active surveillance, surgery or radiotherapy. Tumour size was classified according to Kanzaki et al. as intrameatal, small, moderately large, large or giant tumour [34].

### 2.5 Statistical analysis

Statistical analyses were performed in R version 4.0.5 using Rstudio 1.3.959 (Rstudio, PBC, Boston)

For the demographics and non-responder analysis, means and standard deviation (sd) were calculated for normally distributed numerical variables, and medians and interquartile ranges (IQR) when not normally distributed. For categorical variables, percentages and frequencies were calculated. Demographics of non-responders, responders and one-word responders were compared using the unpaired *t*-test for continuous and chi-squared test for categorical variables. One word responders were defined as patients who provided a one-word answer for all open-ended questions (e.g., “well”, “fine”, or “bad”). Bonferroni correction for multiple testing was used to prevent type-I errors. Incomplete questionnaires were omitted in the analysis.

The ceiling effect, a well-known feature of PREMS, was analysed using the overall experience question of the PEM. In a separate analysis, the outcome of the AI-PREM was evaluated for patients who scored >8 out of 10 on the PEM questionnaire (i.e. provided overall very positive feedback). This analysis was used to assess the capability of the AI-PREM to identify feedback that could be used for quality improvement from patients that were overall positive about their experience with the IPU.

### 2.6 Intervention

The results of the AI-PREM and PEM were used to identify actionable point for quality improvement. The process to analyse, interpret and translate the results are described stepwise. First, results were analysed and placed in the local context by the IPU team. This team, consisting of a deputy of each medical specialism, a researcher, a case manager and supportive staff, used their knowledge of the IPU combined with the PREM results to select feasible and effective projects.

## 3 Results

**Fig. 1:**
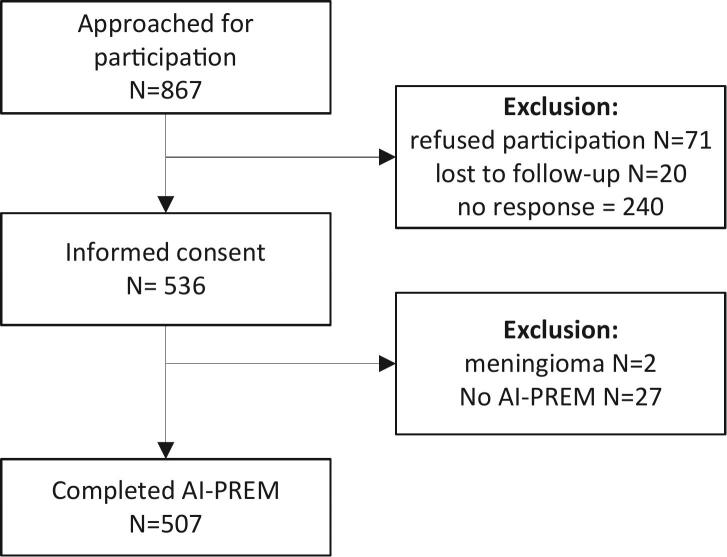
Flowchart study participants.

In total, 536 patients provided informed consent resulting in a 62% response rate, as is shown in Fig. 1. Non-responders more often had a lower level of education (32% vs 44%) but a comparable mean age and male/female ratio to the responders, as shown in Table 1.

**Table 1:** Baseline demographics.

	Non-responders	Not completed	Completed	One-word answers
	*N* = 331	*N* = 28	*N* = 507	*N* = 79
Sex (male)	49%	50%	53%	65%
Age (sd)	68.0(12.3)	69.9 (10.5)	67.4 (11.0)	69.7 (9.6)
Education level				
Low	44%	44%	32%	41%
Middle	25%	33%	30%	27%
High	31%	22%	38%	33%
Treatment				
Observation	61%*	50%	46%	49%
Surgery	26%*	29%	38%	34%
Radiotherapy	13%*	14%	13%	16%
Quality of Life (sd)	69.8 (19.8)*	66.8 (15.5)	69.2 (18.1)	70.4 (17.3)

Demographics are shown for non-responders and responders. Both incomplete and completed questionnaires are shown. One-word responders are a subcategory of completed questionnaires, in which patients completed only one-word answers, such a “good” or “bad”, on each open-ended question. Quality of life shows a disease-specific quality of life questionnaire ranging from 0 to 100. Higher scores indicate better quality of life. sd = standard deviation. * = data acquired in 2014.

Compared to the population of vestibular schwannoma patients, the study population had a somewhat higher mean age (67.4 vs. 61.1 years) as a result of the long term follow-up. Also, the ratio of patients that received active intervention (radiotherapy or surgery) was higher (42% vs 51%), also as a result of the fact that they have been under observation for longer.

### 3.1 AI-PREM outcomes

The AI-PREM was completed by 507 patients, of whom 79 (16%) were one-word responders. As shown in Table 1, one-word responders were more often male, two years older and had a lower education level, but these differences were not statistically significant after correcting for multiple testing. A group of 27 patients did provide informed consent but did not complete the AI-PREM and two patients were excluded because of a pathology different to vestibular schwannoma..

Table 2 shows the different feedback clusters of the five PREM questions including the number of comments per cluster and an example of a raw data comment. The majority of comments was classified as positive. All positive clusters contained many short or monosyllabic responses containing “well” or “fine”, which did not provide additional information or context other than the subject of the question. Negative answers were in general more detailed and contained more words. Due to the diverse nature of the negative feedback, there were more thematic clusters, each containing less individual comments. For example, three negative clusters stated that personal approach was lacking (*N* = 3), limited (N = 3), or insufficient (*N* = 6). Another interesting finding was that different patients may experience certain aspects of care in a contradicting way. Therefore, the number of patients with a positive or a negative experience with the specific aspect of care was quantified, in order to put the feedback into perspective and help decide whether and which action should be taken to improve the IPU. For example, the number of patients who provided positive feedback on scheduling appointments on the same day (*N* = 8) outnumbered those who provided negative feedback on this topic (*N* = 2).

**Table 2:** AI-PREM clusters open-ended question answers.

**Question**	Information provision		Personal approach		Collaboration		Organization		Other experiences	
**Clusters**	*positive*	*negative*	*positive*	*negative*	*positive*	*negative*	*positive*	*negative*	*positive*	*negative*
	Well *n* = 178	Limited* n = 18	Well *n* = 175	Insufficient n = 6	Well *n* = 215	Other hospital n = 5	Well *n* = 205	Appointment* *n* = 34	Well* n = 105	Aftercare *n* = 14
	Clear* n = 178	Lengthy n = 8	Fine *n* = 55	Reserved *n* = 3	Fine *n* = 98	Communication n = 7	Fine *n* = 59	Reachability n = 5	Positive n = 8	Waiting time* n = 21
			Pleasant conversation* *n* = 101	Personal approach n = 3		Bad *n* = 4	Well organized* n = 62			
			Personal approach *n* = 24	Limited n = 3		Suboptimal *n* = 3				
				Support *n* = 4		Better n = 3				
				Experiences* n = 5		Scan n = 9				
				Attention n = 7		Insufficient* n = 8				
**Leftovers**	n = 3	*n* = 0	n = 5	n = 0	n = 10	n = 1	n = 17	n = 0	n = 8	n = 0
Example raw data quotes (from cluster with *)	“The information about the disease and symptoms was clear, informative and understandable.”	“Limited. Several of my symptoms, that were in my opinion related to the tumour, were not addressed at all.”	“Excellent. Understanding and sympathetic about the symptoms. “	“The doctor's ‘empathic capacity’ sometimes did not align with the patient's experiences/feelings.”		“Scheduling a follow-up scan was sometimes difficult.”	“Appointments were mostly scheduled on the same day, which was pleasant.”	“Sometimes you had to wait a long time for the appointment, no appointments scheduled on the same day. Difficult to reach by phone.”	“The vestibular schwannoma team is well-coordinated.”	“Long waiting time for the scan results”

### 3.2 PEM outcomes

The PEM was completed by 490 patients. In general, the patients completed the PEM very positively and the overall experience was graded with an 8 (±1.2 sd) on a 1 to 10 point scale. For example, 95% of the patients trusted their physician, and 93% indicated they had enough time to discuss their problem with the physician. Furthermore, 93% of patients said they discussed what to do after the consultation, and 89% said they were informed about their treatment's pros and cons. The majority (87%) found the physician's explanation understandable. Only 1% indicated they could not ask questions to their consulting physician.

The question with the most negative responses concerned the waiting time in the outpatient clinic. 21% of patients indicated they had to wait >15 min. Of this group, 10% would have preferred to receive more information about the estimated waiting time.

### 3.3 Comparison between PREMs

Table 3 shows the AI-PREM results of patients who scored an overall experience >8 out of 10 points on the PEM questionnaire. These patients had also rather positive experiences on the AI-PREM and only a limited number of negative comments. Still, these comments provided useful and detailed information about the IPU. For example, one patient stated: “I would have liked to hear about the treatment of vertigo with exercises sooner”. Other patients mentioned: “There was some misunderstanding about by whom and when I was called about an appointment.”, “The collaboration between hospitals was poor.”, and “I was discharged from the hospital too soon and without instructions.”

**Table 3:** AI-PREM results of patients with an overall PEM scores of >8/10.

	Negative		Neutral		Positive	
	count	%	count	%	count	%
Information provision	3	2%	37	23%	122	75%
Personal approach	2	1%	35	22%	125	77%
Collaboration	6	4%	35	22%	121	75%
Organization	6	4%	35	22%	121	75%
Other experiences	7	4%	90	56%	65	40%

### 3.4 Observation of the interpretations of results

The results of the close-ended PEM questionnaire were predominantly positive, which was considered motivating information for the IPU team. However, for quality improvement these positive reactions could not be translated to action points for improvement. Conversely, the AI-PREM results provided more detailed information about the positive and negative experiences, even from patients that provided overall positive feedback. This information could be used to identify action points.

**Fig. 2:**
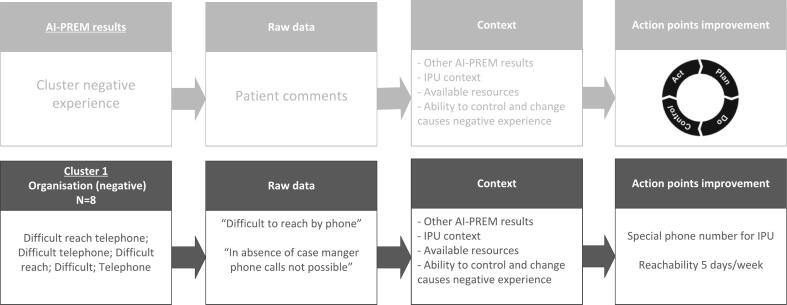
Process from AI-PREM results to quality improvement. The process steps from using the AI-PREM results to identify action points for quality improvement are shown in grey. The second row shows the process steps of the identified action point reachability by phone.

The process to identify action points for improvement is shown in Fig. 2. First, the IPU team analysed the results of the AI-PREM and explored the negative clusters of patients' experiences for potential quality improvements. The automated sentiment analysis and clustering of comments was used to identify topics of interest. These topics of interest were subsequently further explored by the IPU team through targeted evaluation of clustered patient comments (raw text). These raw texts were valued in the context of the IPU organization. When potential action points emerged they were discussed in the meeting and weighed against possible positive feedback regarding the same topic.

In all, the IPU team selected three action points for quality improvement based on actionability, feasibility and number of patients sharing the particular (negative) experience. The chosen action points were improving the reachability by phone, reducing the time between the MRI and the consultation to discuss the result and improving the communication with referring hospitals.

## 4 Discussion and conclusion

### 4.1 Discussion

To our knowledge, this is the first study in which a PREM with open-ended questions is directly compared to a traditional PREM with close-ended questions. Both questionnaires allowed evaluation of patient experiences with the care provided by the vestibular schwannoma care pathway. Both questionnaires reported overall positive patients' experiences.

The PEM enabled an easy and quick quantitative analysis of the overall experience. Most results showed ceiling effects and the predefined answer categories were less suited for identification of points of improvement, especially in the context of predominantly positive experiences. The AI-PREM seemed to have a greater potential to identify actionable points for quality improvement because of the broader focus and the more detailed descriptions, especially of negative experiences. With the AI-PREM, feedback with improvement points could be obtained even from patients with very positive experiences (as judged on the PEM scores).

An essential feature determining feasibility for clinical use was the automated analysis of the open text PREMs to reduce the workload. Still, the human component in the analysis is essential to interpret the algorithm's results and combine this with the clinical context of the IPU to translate the feedback into actionable points of improvement. Furthermore, the AI-PREM combined output of quantitative and more qualitative data. This combination of sentiment scores, the number of comments per cluster and a traceback to the individual reported experience facilitated decision making for quality improvement. In contrast, the use of the structured PEM for identification of points of improvement was limited due to a small number of reported negative experiences.

The AI-PREM results showed that most comments were positive, but negative comments provided more detailed descriptions, including more context. Positive comments were more often one-word answers and generic. These findings were also described by Cunningham et al. while analysing almost 7000 open-text comments [22]*.* Positive comments are essential to put the negative ones into context and prioritize action points for improvement. For example, when many comments are positive about scheduling appointments, some negative comments on this cluster might be outliers, making this a less urgent target for quality improvement. In addition, positive comments can be used as motivators for the IPU team and can contribute to increasing patient safety following the Safety-II paradigm, which focuses on the things that go right rather than focusing on things that go wrong [16,35]*.*

Other studies, focussing on patients narratives, have reported that the patients' comments on their experience with disease and care delivery generally provide mainly positive outcomes [16,17,36]. For example, the study of De Rosis et al. reported mainly positive comments which could be used for to identify positive aspects, which could be used for quality improvement by a ‘learning by excellence’ strategy. While this is valuable, learning by excellence in itself has a limited ability to to identify actionable points for improvement. The AI-PREM presented here has the ability to show and quantify positive comments but at the same time identify points of improvement, even in the feedback of patients that are overall positive about their experience in the IPU. In doing so, a more nuanced feedback of patients on the care delivery is made possible. While we find, like previous reports, that a large majority of patients provide positive comments, we were also able to extract actionable points of improvement even from patients with generally positive feedback.

Also in research settings, generic PREMs are used to evaluate the quality improvement targeted at improving the overall patient experience [36]*.* Improving organizational factors for a better patient experience will not only benefit patients but has also been shown to enhance physician satisfaction [37]*.* However, achieving improvements in the patient experience can be challenging [38]*.* A large proportion of patients report high PREM scores. This ceiling effect might be caused by appreciation or social desirability bias [39,40]*.* In this study, the PEM results also show this ceiling effect, which is challenging from a quality improvement perspective since these already high scores can be hard to improve on. When trying to improve patient care, focussing on overall patient satisfaction or PREM scores may therefore be less effective than evaluating the negative comments in detail. Moreover, this study shows that even patients with a positive overall experience (as reported in the PEM) may still have feedback indicating points of improvement (identified with the AI-PREM). The AI-PREM design allows for an in-depth analysis of the comments by grouping them together in clusters based on sentiment and similar word content. Consequently, the actual remarks concerning a certain topic made by individual patients can be accessed, providing all necessary detail, without manually going through all questionnaires to extract information about the topic at hand. This approach, which yields both quantitative and qualitative data from free-text answers, saves time yet allows patients to comment freely on their experience with all aspects of care, detailed analysis of their feedback and identification of specific points of improvement.

A potential problem of using PREMs for quality improvements is a selection bias of the patients who complete the PREMs. When the responders are not a random sample of the total patient population the risk for inadequately aimed quality optimisations exists. Younger patients and black, indigenous and people of colour tend to report less positive patient experiences [41,42]*.* So it is important to include answers of these groups in the analysis for quality improvement. The non-responder analysis showed a larger proportion of lower education level in this group. There were no age differences, but one-word responders were on average slightly elder. These aspects should be considered when interpreting the PREM results to prevent nonresponse errors [43]*.*

In addition, open-ended PREMs might reflect the a priori expectations and perceptions of care. When the provided care meets the expectations, patients might not provide feedback but they probably will when the experience is worse or much better than their expectations. This phenomenon is especially important since different populations have different expectations of care delivery [44,45]*.* The evolution from patient satisfaction (e.g., how would you rate the information you received about your treatment?) towards experience (e.g., did you receive information about your treatment?) has mitigated the risk of such bias [45]*.* However, open-ended questions in structured PREMs are often focussed on patient satisfaction (e.g., “What went remarkably well during your stay?”). The AI-PREM questions focus more on the experience and reduce but not neutralize the risk of expectation bias.

In this study, a patient population was selected that had already participated in previous research. These dedicated participants might introduce some selection bias. When collecting the PREMs prospectively, the response rate might, therefore, be lower. Another limitation was the prolonged recall period since the last visit to the hospital in this research. The period exceeded the 4–6 weeks used in the PEM validation study [14]*.* This prolonged period might have limited the output of the PREMs [2]*.* However, the comparison between the two PREMS was not affected since both questionnaires were completed simultaneously.

### 4.2 Experiences of deployment in a vestibular schwannoma IPU

The IPU team used the PREM results to identify actionable points for quality improvement. This entailed a process of interpretation of the PREM results and analysing them in order to use them to improve clincal practice. Important parameters during the IPU team discussions were the quantitative results and the positive feedback clusters. The quantitative information (how many patients shared the same view) was useful in determining the extent of the problem. However, the positive feedback was essential too, for putting certain negative comments into perspective and prioritizing and focusing actions on improving the care delivery. Taking action based on the negative comments only could mistakenly alter aspects of care that provided a positive experience for most patients.

In addition, the potential of the IPU to improve or change the underlying causes of the negative experience was discussed. For example, a negative patient experience about a lack of parking space is beyond the control of the IPU, but the communication about the appointments is within the sphere of influence of the IPU. When potential action points were within the sphere of influence, the available resources needed to perform an improvement cycle were identified to see whether an improvement cycle was feasible. Finally, the IPU team decided to start a plan, do, check, act cycle.

### 4.3 Innovation

With the growing interest in patient-centeredness of care comes a growing need to adequately assess the patient experience with care delivery. The AI-PREM may be a tool that allows patients to freely comment on their experience yet is economic with the time and effort invested by healthcare professionals to analyse the feedback, although the time and effort invested by patients to complete the AI-PREM should also be considered. To make the efforts of patients worthwhile, PREMs should be used to improve care delivery, rather than as an administrative requirement. Future research should evaluate the applicability of the AI-PREM in different clinical settings. Because of the generic nature of the AI-PREM questionnaire, it seems likely to be of value in a multitude of different diseases, care pathways, or healthcare centres. In addition, the ability of the AI-PREM to detect longitudinal changes in the quality of care and/or the effect of measures to improve the quality of care may be the subject of future research.

### 4.4 Conclusion

Patient experiences are an essential aspect of quality of care. This study showed the added value of open-ended PREM questions in assessing patient experiences. The AI-PREM provided insights into both positive and negative experiences and allowed the detection of actionable targets for quality improvement in an IPU. Because of its automated analysis and readily accessible results, the evaluation of the patient experience with the vestibular schwannoma care pathway could be performed by IPU clinicians and translated into action points relevant to context of the clinical IPU.

## Funding

This research did not receive any specific grant from funding agencies in the public, commercial, or not-for-profit sectors.

## CRediT authorship contribution statement

**O.M. Neve:** Conceptualization, Methodology, Investigation, Formal analysis, Writing – original draft. **M.M. van Buchem:** Data curation, Investigation, Software, Writing – review & editing. **M. Kunneman:** Writing – review & editing. **P.P.G. van Benthem:** Writing – review & editing. **H. Boosman:** Conceptualization, Methodology, Writing – review & editing. **E.F. Hensen:** Conceptualization, Supervision, Writing – original draft.

## Declaration of Competing Interest

The authors declare that they have no known competing financial interests or personal relationships that could have appeared to influence the work reported in this paper.

## References

[1] Department of Health, D.o. Health (Ed.) (2008). High quality care for all: NHS next stage review final report.

[2] Manary M.P., Boulding W., Staelin R., Glickman S.W. (2013). The patient experience and health outcomes. N Engl J Med.

[3] Gleeson H., Calderon A., Swami V., Deighton J., Wolpert M., Edbrooke-Childs J. (2016). Systematic review of approaches to using patient experience data for quality improvement in healthcare settings. BMJ Open.

[4] Bull C., Byrnes J., Hettiarachchi R., Downes M. (2019). A systematic review of the validity and reliability of patient-reported experience measures. Health Serv Res.

[5] Greaves F., Jha A.K. (2014). Quality and the curate's egg. BMJ Qual Saf.

[6] Black N., Varaganum M., Hutchings A. (2014). Relationship between patient reported experience (PREMs) and patient reported outcomes (PROMs) in elective surgery. BMJ Qual Saf.

[7] Doyle C., Lennox L., Bell D. (2013). A systematic review of evidence on the links between patient experience and clinical safety and effectiveness. BMJ Open.

[8] Rivara M.B., Edwards T., Patrick D., Anderson L., Himmelfarb J., Mehrotra R. (2021). Development and content validity of a patient-reported experience measure for home dialysis. Clin J Am Soc Nephrol.

[9] Zinckernagel L., Schneekloth N., Zwisler A.-D.O., Ersbøll A.K., Rod M.H., Jensen P.D. (2017). How to measure experiences of healthcare quality in Denmark among patients with heart disease? The development and psychometric evaluation of a patient-reported instrument. BMJ Open.

[10] Taylor R.M., Fern L.A., Solanki A., Hooker L., Carluccio A., Pye J. (2015). Development and validation of the BRIGHTLIGHT Survey, a patient-reported experience measure for young people with cancer. Health Qual Life Outcomes.

[11] Bosworth A., Cox M., O’Brien A., Jones P., Sargeant I., Elliott A. (2015). Development and validation of a patient reported experience measure (PREM) for patients with rheumatoid arthritis (RA) and other rheumatic conditions. Curr Rheumatol Rev.

[12] Bobrovitz N., Santana M., Kline T., Kortbeek J., Stelfox H.T. (2013). Prospective cohort study protocol to evaluate the validity and reliability of the Quality of Trauma Care Patient-Reported Experience Measure (QTAC-PREM). BMC Health Serv Res.

[13] Jenkinson C. (2002). The Picker Patient Experience Questionnaire: development and validation using data from in-patient surveys in five countries. International J Qual Health Care.

[14] Bastemeijer C.M., Boosman H., Zandbelt L., Timman R., De Boer D., Hazelzet J.A. (2020). Patient experience monitor (PEM): the development of new short-form picker experience questionnaires for hospital patients with a wide range of literacy levels. Patient Related Outcome Measures.

[15] Giordano L.A., Elliott M.N., Goldstein E., Lehrman W.G., Spencer P.A. (2010). Development, implementation, and public reporting of the HCAHPS survey. Med Care Res Rev.

[16] De Rosis S., Cerasuolo D., Nuti S. (2020). Using patient-reported measures to drive change in healthcare: the experience of the digital, continuous and systematic PREMs observatory in Italy. BMC Health Serv Res.

[17] Corazza I., Gilmore K.J., Menegazzo F., Abols V. (2021). Benchmarking experience to improve paediatric healthcare: listening to the voices of families from two European Children’s University Hospitals. BMC Health Serv Res.

[18] Decourcy A., West E., Barron D. (2012). The National Adult Inpatient Survey conducted in the English National Health Service from 2002 to 2009: how have the data been used and what do we know as a result?. BMC Health Serv Res.

[19] Coulter A., Locock L., Ziebland S., Calabrese J. (2014). Collecting data on patient experience is not enough: they must be used to improve care. BMJ.

[20] Davies E. (2005). Hearing the patient's voice? Factors affecting the use of patient survey data in quality improvement. Qual Saf Health Care.

[21] Kunneman M., Montori V.M., Shah N.D. (2017). Measurement with a wink. BMJ Qual Saf.

[22] Cunningham M., Wells M. (2017). Qualitative analysis of 6961 free-text comments from the first National Cancer Patient Experience Survey in Scotland. BMJ Open.

[23] Riiskjaer E., Ammentorp J., Kofoed P.E. (2012). The value of open-ended questions in surveys on patient experience: number of comments and perceived usefulness from a hospital perspective. International J Qual Health Care.

[24] Garcia J., Evans J., Reshaw M. (2004). “is there anything else you would like to tell us” – methodological issues in the use of free-text comments from postal surveys. Qual Quant.

[25] Cammel S.A., De Vos M.S., Van Soest D., Hettne K.M., Boer F., Steyerberg E.W. (2020). How to automatically turn patient experience free-text responses into actionable insights: a natural language programming (NLP) approach. BMC Med Inform Decis Mak.

[26] Arditi C., Walther D., Gilles I., Lesage S., Griesser A.-C., Bienvenu C. (2020). Computer-assisted textual analysis of free-text comments in the Swiss Cancer Patient Experiences (SCAPE) survey. BMC Health Serv Res.

[27] Van Buchem M.M., Neve O.M., Kant I.M.J., Steyerberg E.W., Boosman H., Hensen E.F. (2022). Analyzing patient experiences using natural language processing: development and validation of the artificial intelligence patient reported experience measure (AI-PREM). BMC Med Inform Decis Mak.

[28] Carlson M.L., Link M.J. (2021). Vestibular Schwannomas. N Engl J Med.

[29] Soulier G., Van Leeuwen B.M., Putter H., Jansen J.C., Malessy M.J.A., Van Benthem P.P.G. (2017). Quality of life in 807 patients with vestibular schwannoma: comparing treatment modalities. Otolaryngology–Head and Neck Surgery.

[30] Gerteis M., Edgman-Levitan S., Daley J., Delbanco T.L. (1993).

[31] van Leeuwen B.M., Herruer J.M., Putter H., Jansen J.C., van der Mey A.G., Kaptein A.A. (2013). Validating the Penn Acoustic Neuroma Quality Of Life Scale in a sample of Dutch patients recently diagnosed with vestibular schwannoma. Otol Neurotol.

[32] Shaffer B.T., Cohen M.S., Bigelow D.C., Ruckenstein M.J. (2010). Validation of a disease-specific quality-of-life instrument for acoustic neuroma. Laryngoscope.

[33] Statistics Netherlands (2017).

[34] Kanzaki J., Tos M., Sanna M., Moffat D.A., Monsell E.M., Berliner K.I. (2003). New and modified reporting systems from the consensus meeting on systems for reporting results in vestibular schwannoma. Otol Neurotol.

[35] Braithwaite J., Wears R.L., Hollnagel E. (2015). Resilient health care: turning patient safety on its head. International J Qual Health Care.

[36] Bastemeijer C.M., Boosman H., Van Ewijk H., De Jong-Verweij L.M., Voogt L., Hazelzet J. (2019). Patient experiences: a systematic review of quality improvement interventions in a hospital setting. Patient Related Outcome Measures.

[37] Golda N., Beeson S., Kohli N., Merrill B. (2018). Analysis of the patient experience measure. J Am Acad Dermatol.

[38] Wong E., Mavondo F., Horvat L., McKinlay L., Fisher J. (2021). Victorian healthcare experience survey 2016–2018; evaluation of interventions to improve the patient experience. BMC Health Serv Res.

[39] Kleiss I.I., Kortlever J.T., Karyampudi P., Ring D., Brown L.E., Reichel L.M. (2020). A comparison of 4 single-question measures of patient satisfaction. J Clin Outcomes Manag.

[40] Salman A.A., Kopp B.J., Thomas J.E., Ring D., Fatehi A. (2020). What are the priming and ceiling effects of one experience measure on another?. J Patient Exp.

[41] Campbell J.L. (2001). Age, gender, socioeconomic, and ethnic differences in patients’ assessments of primary health care. Qual Health Care.

[42] Lyratzopoulos G., Elliott M., Barbiere J.M., Henderson A., Staetsky L., Paddison C. (2012). Understanding ethnic and other socio-demographic differences in patient experience of primary care: evidence from the English General Practice Patient Survey. BMJ Qual Saf.

[43] Johnson T.P. (2012). Response rates and nonresponse errors in surveys. JAMA.

[44] Poole K.G. (2019). Patient-experience data and Bias — what ratings Don’t tell us. N Engl J Med.

[45] Ahmed F., Burt J., Roland M. (2014). Measuring patient experience: concepts and methods. The Patient - Patient-Centered Outcomes Research.

